# Supplementary material for: Nitrated Graphene Oxide Derived from Graphite Oxide: A Promising Energetic Two-Dimensional Material
Source: Nanomaterials (Basel). 2020 Dec 29;11(1):58. doi: 10.3390/nano11010058 (PMC7823779; doi:10.3390/nano11010058)
Supplement: Supplementary file 1 [file nanomaterials-11-00058-s001.pdf]

Supplement information

Article

Nitrated Graphene Oxide Derived from Graphite Oxide: A Promising Energetic Two-Dimensional Material

Fayang Guan [1], Hui Ren [1,*], Lan Yu [2], Qingzhong Cui [1], Wanjun Zhao [1] and Jie Liu [1]

[1] State Key Laboratory of Explosion Science and Technology, Beijing Institute of Technology, Beijing 100081, China; casguan@163.com (F.G.); cqz1969@bit.edu.cn (Q.C.); wanjunzhaowj@gmail.com (W.Z.); liujie0417@bit.edu.cn (J.L.)

[2] Hong Kong New ARK Technologise LTD, Hong Kong 999077, China; gutti14@126.com

* Correspondence: renhui@bit.edu.cn;

Table S1. Bond length and angles of the stable configuration when the central epoxy and nitroxyl cation are at different distances

$L(N \cdot\cdot O_3)$	$\angle C_1O_3C_2$	$L(C_1\text{-}O_3)$	$L(C_2\text{-}O_3)$	$L(C_1\text{-}C_2)$	$\angle O_1NO_2$	$\angle O_1NO_3$	$\angle O_2NO_3$	$L(O_1\text{-}N)$	$L(O_2\text{-}N)$	Coplanarity ($NO_1O_2O_3$)
3.43	68.70	1.43	1.43	1.62	133.08	72.69	66.44	1.21	1.21	272.21
2.86	68.23	1.43	1.44	1.61	133.34	85.94	85.57	1.21	1.21	304.85
2.47	68.10	1.44	1.44	1.61	133.16	98.66	102.76	1.21	1.21	334.58
2.29	67.69	1.44	1.44	1.60	132.84	104.96	102.54	1.21	1.21	340.34
1.93	63.73	1.48	1.48	1.57	131.66	107.47	109.15	1.21	1.22	348.28
1.84	44.40	2.18	1.47	1.53	134.74	107.64	117.58	1.19	1.20	359.96
1.65	28.30	2.73	3.04	1.44	132.25	114.57	113.19	1.21	1.21	360.00
1.46	10.63	5.78	4.71	1.44	127.25	115.30	117.45	1.22	1.23	359.99

*The unit of length is Ångstrom(Å) and the angle unit is degree(°). Coplanarity is the sum of angles with N as the vertex.

Table S2. Bond length and angles of the stable configuration when the marginal epoxy and nitroxyl cation are at different distances

$L(N{\cdot\cdot}O_4)$	$\angle C_3O_4C_4$	$L(C_3\text{-}O_4)$	$L(C_4\text{-}O_4)$	$L(C_3\text{-}C_4)$	$\angle O_1NO_2$	$\angle O_1NO_4$	$\angle O_2NO_4$	$L(O_1\text{-}N)$	$L(O_2\text{-}N)$	Coplanarity ($NO_1O_2O_4$)
3.41	53.86	1.48	1.45	1.32	133.15	92.26	93.97	1.21	1.21	319.38
2.75	53.82	1.48	1.45	1.32	133.20	92.40	94.34	1.21	1.21	319.94
2.46	53.73	1.48	1.45	1.32	132.83	99.47	100.75	1.21	1.21	333.05
2.20	53.66	1.48	1.45	1.32	131.66	102.31	106.10	1.21	1.22	340.06
1.91	53.18	1.50	1.45	1.32	130.52	106.64	107.31	1.22	1.22	344.48
1.85	53.03	1.51	1.46	1.32	130.85	106.81	107.31	1.22	1.22	344.97
1.63	29.28	2.39	1.36	1.37	134.90	110.13	114.97	1.19	1.20	360.00
1.42	29.53	2.39	1.40	1.36	131.02	111.71	117.27	1.21	1.21	360.00

*The unit of length is Ångstrom(Å) and the angle unit is degree(°). Coplanarity is the sum of angles with N as the vertex.

Table S3. Bond length and angles of the stable configuration when the marginal hydroxyl and nitroxyl cation are at different distances

L(N··O_5)	∠O_1NO_2	∠O_1NO_5	∠O_2NO_5	L(O_1-N)	L(O_2-N)	L(O_5-H_1)	L(O_5-C_5)
3.42	133.13	84.52	88.97	1.19	1.21	0.960	1.35
2.89	133.34	84.20	87.70	1.21	1.21	0.978	1.36
2.42	133.13	97.86	102.97	1.21	1.21	0.979	1.36
2.16	132.23	100.14	104.86	1.21	1.21	0.980	1.36
1.92	131.13	103.40	106.43	1.22	1.22	0.982	1.37
1.86	131.58	104.30	107.33	1.22	1.22	0.983	1.38
L(N··C_5)	∠O_1NC_5	∠O_2NC_5	Coplanarity ($NO_1O_2C_5$)		Coplanarity ($NO_1O_2O_5$)		
4.56	94.48	85.07	312.68		306.63		
4.00	96.04	83.54	312.92		305.24		
3.58	104.14	89.84	327.12		333.96		
3.29	116.40	90.45	339.08		337.24		
2.85	128.05	86.81	345.99		340.96		
2.83	128.63	85.91	346.11		343.21		

*The unit of length is Ångstrom(Å) and the angle unit is degree(°). Coplanarity is the sum of angles with N as the vertex.

Table S4. Bond length and angles of the stable configuration when the central hydroxyl and nitroxyl cation are at different distances

$L(N\cdot\cdot O_6)$	$\angle O_1NO_2$	$\angle O_1NO_6$	$\angle O_2NO_6$	$L(O_1\text{-}N)$	$L(O_2\text{-}N)$	$L(O_6\text{-}H2)$	$L(O_6\text{-}C_6)$
3.29	132.43	95.04	106.60	1.21	1.21	0.976	1.48
2.88	132.34	96.13	110.12	1.21	1.21	0.977	1.48
2.47	131.08	94.86	109.98	1.21	1.21	0.976	1.47
2.16	130.80	99.64	106.16	1.21	1.21	0.978	1.47
1.99	127.59	105.09	106.53	1.23	1.22	0.980	1.51
1.57	126.19	110.73	109.12	1.25	1.23	0.978	2.59

$L(N\cdot\cdot C_6)$	$\angle O_1NC_6$	$\angle O_2NC_6$	Coplanarity ($NO_1O_2C_6$)	Coplanarity ($NO_1O_2O_6$)
4.01	106.73	107.69	346.86	334.08
3.66	108.06	111.03	351.43	338.58
2.91	106.61	116.15	353.83	335.92
2.74	110.54	113.71	355.05	336.60
2.13	115.52	116.17	359.28	339.22
1.86	104.81	103.96	334.96	346.04

*The unit of length is Ångstrom(Å) and the angle unit is degree(°). Coplanarity is the sum of angles with N as the vertex.
